# Supplementary material for: Pharmacokinetic/Pharmacodynamic Evaluation of Aztreonam/Amoxicillin/Clavulanate Combination against New Delhi Metallo-β-Lactamase and Serine-β-Lactamase Co-Producing Escherichia coli and Klebsiella pneumoniae
Source: Pharmaceutics. 2023 Jan 11;15(1):251. doi: 10.3390/pharmaceutics15010251 (PMC9865866; doi:10.3390/pharmaceutics15010251)
Supplement: Supplementary file 1 [file pharmaceutics-15-00251-s001.zip › pharmaceutics-2141590-supplementary.pdf]

Supplemental material for

Pharmacokinetic/Pharmacodynamic Evaluation of Aztreonam/Amoxicillin/Clavulanate Combination against New Delhi Metallo-β-Lactamase and Serine-β-Lactamase Co-Producing *Escherichia coli* and *Klebsiella pneumoniae*

Jiayuan Zhang [1], Mengyuan Wu [1], Shuo Diao [1], Shixing Zhu [1], Chu Song [1], Jiali Yue [1], Frederico S. Martins [2], Peijuan Zhu [3], Zhihua Lv [1,4,*], Yuanqi Zhu [5], Mingming Yu [1,4,*] and Sherwin K. B. Sy [6]

[1] School of Medicine and Pharmacy, Ocean University of China, 5 Yushan Road, Qingdao 266003, China
[2] Faculty of Pharmaceutical Sciences of Ribeirão Preto, University of São Paulo, Ribeirão Preto 14040-900, São Paulo, Brazil
[3] Department of Pharmacology, University of Pennsylvania, Philadelphia, Pennsylvania 19104, USA
[4] Laboratory for Marine Drugs and Bioproducts of Qingdao National Laboratory for Marine Science and Technology, Qingdao 266003, China
[5] Department of Laboratory Medicine, The Affiliated Hospital of Qingdao University, Qingdao 266003, China
[6] Department of Statistics, State University of Maringá, Maringá 87020-900, Paraná, Brazil
* Correspondence: lvzhihua@ouc.edu.cn (Z.L.); yumingming@ouc.edu.cn (M.Y.); Tel.: +86-17852411792 (M.Y.)

Methods

Detailed description of pharmacokinetic models

Body weight (WT) and creatinine clearance (CrCL) were influential covariates of the model parameters. Creatinine clearance was a uniform distribution from 10 to 150 mL/min. Body weights of males and females were derived [46,47] from height distributions of male ($176.3 \pm 0.17\sqrt{4482}$ cm (mean ± SD)) and female ($162.2 \pm 0.16\sqrt{4857}$) in cm [48], such that

$$WT_{male} = \exp(3.28 + 1.92 \log HT_{male}) \exp(\omega_m) \text{ where } \omega_m \sim N(0,0.14)$$

$$WT_{female} = \exp\left(3.49 + 1.45 \log HT_{female}\right) \exp\left(\omega_f\right) \text{ where } \omega_f \sim N(0,0.17)$$

A two-compartment intravenous model was defined by the following ordinary differential equations:

$$\frac{dA_1}{dt} = -A_1(CL/V_C + Q/V_C) + A_2(Q/V_P)$$

$$\frac{dA_2}{dt} = A_1(Q/V_C) - A_2(Q/V_P)$$

where CL (L/h) refers to clearance, Q (L/h) for intercompartmental clearance, V_C (L) for central volume, V_P (L) for peripheral volume, A_1 is the drug amount in the central compartment and A_2 is the drug amount in the peripheral compartment. Systemic total drug concentration is obtained by dividing A_1 at time t by V_C. The unbound (or free) drug concentration is obtained by multiplying total drug concentration by (1 – protein binding fraction).

For amoxicillin, the individual pharmacokinetic parameters [27] for were as follow:

$$CL_i = 10.3(CrCL/102) \exp(\omega_{CL}) \text{ where } \omega_{CL} \sim N(0, 0.399)$$

$$V_{C,i} = 13.5 \exp(\omega_{Vc}) \text{ where } \omega_{Vc} \sim N(0,0.387)$$

$$V_P = 14.1$$

$$Q = 15.7$$

The individual pharmacokinetic parameters for clavulanate [27] were as follow:

$$CL_i = 6.8(CrCL/102) \exp(\omega_{CL}) \text{ where } \omega_{CL} \sim N(0, 0.578)$$

$$V_{C,i} = 7.6 \exp(\omega_{Vc}) \text{ where } \omega_{Vc} \sim N(0,0.347)$$

$$V_P = 11.6$$

$$Q = 10.4$$

For aztreonam, the individual parameters [25] were defined as follow:

$$CL_i = 4.93(CrCL/100)^{0.43} \exp(\omega_{CL}) \text{ where } \omega_{CL} \sim N(0, 0.241)$$

$$V_{C,i} = 7.43(WT/70)^{1.99} \exp(\omega_{Vc}) \text{ where } \omega_{Vc} \sim N(0,0.509)$$

$$V_P = 6.44 \exp\left(\omega_{Vp}\right) \text{ where } \omega_{Vp} \sim N(0, 0.277)$$

$$Q = 9.26$$

A sample RxODE script for simulating aztreonam concentration-time profiles is shown below:

```
m1 <- RxODE({
  A1 = centr;
  A2 = peri;
  d/dt(centr) = - A1*(CL/V1 + Q/V1) + A2*Q/V2;
  d/dt(peri)  =   A1*Q/V1 - A2*Q/V2;
})

sim <- function(...){
    ev <- getEv(...)
    par <- data.frame(WT=WT, CrCL=CrCL,
                      CL=4.93*(CrCL/100)^0.43*exp(rnorm(length(CrCL), mean=0, sd=0.241)),
                      V1=7.43*(WT/70)^1.99*exp(rnorm(length(WT), mean=0, sd=0.509)),
                      Q=9.26,
                      V2=6.44*exp(rnorm(length(WT), mean=0, sd=0.077)))
    rxSolve(m1, par, ev)
}

# init values for first and second compartments
inits <- c(0, 0)
# define time for sampling
s1 <- c(seq(0,4,by=0.1),4.25,4.5,4.75,5,5.25,5.5,5.75)
s2<-c(s1,s1+6,s1+6*2,s1+6*3,s1+6*4,s1+6*5,s1+6*6,s1+6*7,s1+6*8,s1+6*9,s1+6*10,s1+6*11,6*12)

# define covariates
nsub=10000 # set-up for the number of individuals in the simulation
SEX<-round(runif(nsub,min=0,max=1))
HTm<-round(rnorm(nsub,176.3,0.17*sqrt(4482)),digits=1)
HTf<-round(rnorm(nsub,162.2,0.16*sqrt(4857)),digits=1)
WTm<-round(exp(3.28+1.92*log(HTm/100))*exp(rnorm(nsub,0,0.14)),digits=1)
WTf<-round(exp(3.49+1.45*log(HTf/100))*exp(rnorm(nsub,0,0.17)),digits=1)
WT<-ifelse(SEX==1,WTf,WTm)
# define creatinine clearance by renal function categories
CrCL0<-round(runif(nsub,min=51, max=150))
CrCL1<-round(runif(nsub,min=151, max=190))
CrCL2<-round(runif(nsub,min=31, max=50))
CrCL3<-round(runif(nsub,min=10, max=30))

# define model parameters
theta.aztreonam <-
  cbind(WT,CrCL=CrCL0,
        CL=4.93*(CrCL0/100)^0.43*exp(rnorm(nsub, mean=0, sd=0.241)),
        V1=7.43*(WT/70)^1.99*exp(rnorm(nsub,0,.509)),
        Q=9.26,
        V2=6.44*exp(rnorm(length(WT), mean=0, sd=0.277)))

nobs = length(s2) # number of observation is defined as the number of sampling time
cp.aztreonam = matrix(NA, nobs, nsub)

for (i in 1:nsub)
{
  print(i)
  theta = theta.aztreonam[i,]
  ev<-eventTable()
  ev$add.dosing(dose=2000, nbr.doses=1, rate=2000/3, start.time=0)
  ev$add.dosing(dose=1500, nbr.doses=12, rate=1500/3, start.time=6, dosing.interval=6)
  ev$add.sampling(s2)
  conc <- m1$run(theta, ev, inits=inits)
  cp.aztreonam[, i] = conc[, "A1"] /theta.aztreonam[i,"V1"]
}

cp.aztreonam<-as.data.frame(cp.aztreonam)
cp.aztreonam$time=s2

res<-melt(cp.aztreonam, id.vars=c('time'))

res$free<-res$value*(1-0.56)  # Aztreonam has 56% protein binding

# compute summary statistics for plotting
freesum<-ddply(res, 'time', summarise,
               q025 = quantile(free, 0.025),
               q500 = quantile(free, 0.50),
               q975 = quantile(free, 0.975))

# define labels
z00<-expression(bold('Time (h)'))
z01<-expression(bold(paste('Free aztreonam (',mu,'g/mL)')))
```

```
z02<-expression(bold(paste('Aztreonam breakpoint: 8 ',mu,'g/mL')))
z03<-expression(bold('Aztreonam in CrCL >50-130 mL/min'))

# create plot
p1<-ggplot(freesum)+
  geom_ribbon(aes(x=time, ymin=q025, ymax=q975), fill='grey70')+
  geom_line(aes(x=time, y=q500))+
  geom_hline(yintercept=8, linetype=2, color='grey80') +
  annotate('text',x=36, y=7.2, label=z02)+
  xlab(z00) + ylab(z01)+ ggtitle(z03) +
  scale_x_continuous(breaks=c(seq(0,72,by=3)))+
  scale_y_log10(breaks=c(0.125,0.25,0.5,1,2,4,8,16,32,64),
              labels=c(0.125,0.25,0.5,1,2,4,8,16,32,64)) +
  coord_cartesian(ylim=c(0.1,100)) +
  theme_bw() +
  theme(panel.grid.minor = element_blank())

p1
```

Derivation of pharmacodynamic parameters

From each individual free drug concentration time profile, the pharmacodynamic (PD) parameters are derived by computing the time above mutant prevention concentration ($fT_{>MPC}$) and time above minimum inhibitory concentration ($fT_{>MIC}$), as illustrated in Figure S1. The time at which drug concentrations intersected with the MIC and MPC when free drug concentration is increasing and decreasing over time was determined by interpolation. The time at which free drug concentration is within the mutant selection window (fT_{MSW}) was determined by substracting $fT_{>MIC}$ from $fT_{>MPC}$. The shaded area in Figure S1 represents fT_{MSW}.

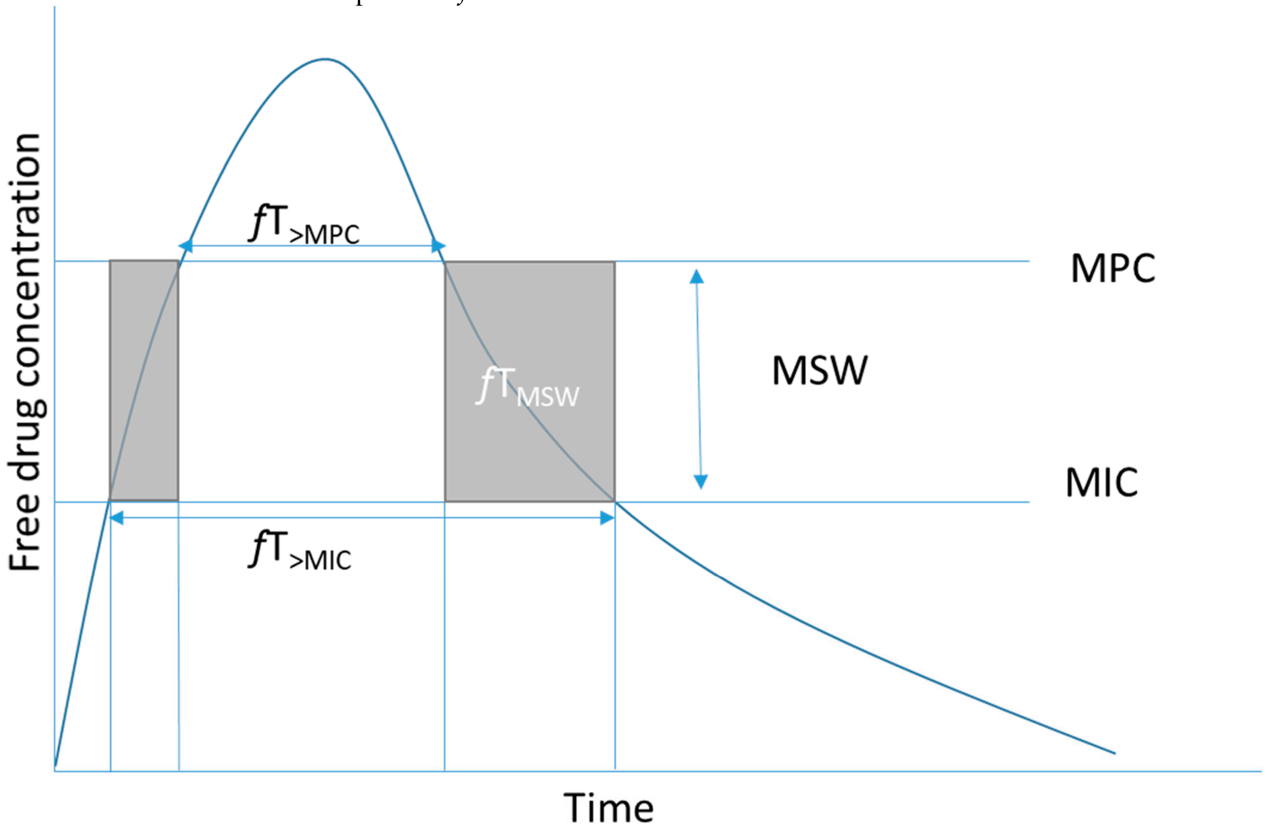

Figure S1. Illustration of derivation of pharmacodynamic indices for suppression of mutant selection. Abbreviations: MIC, minimum inhibitory concentration; MPC, mutant prevention concentration; MSW, mutant selection window; $fT_{>MIC}$, fraction of time that free drug concentration is above MIC; $fT_{>MPC}$, fraction of time that free drug concentration is above MPC; fT_{MSW}, fraction of time wherein free drug concentration is within MSW.

Computation of probability of target attainment

The β-lactams are time-dependent killing antibiotics. Their PD indices are best-described by $fT_{>MIC}$ [49]. The target values for these PD indices are obtained from the literature. From 10,000 simulated free drug concentration-time profiles, the proportion from 10,000 profiles wherein $fT_{>MIC}$ is at least the target PD index for the specific MIC value is defined as the probability of target attainment. This probability is determined for each incremental MIC.
